# The Correlation between Physical Crosslinking and Water-Soluble Drug Release from Chitosan-Based Microparticles

**DOI:** 10.3390/pharmaceutics12050455

**Published:** 2020-05-16

**Authors:** Emilia Szymańska, Katarzyna Woś-Latosi, Julia Jacyna, Magdalena Dąbrowska, Joanna Potaś, Michał Jan Markuszewski, Katarzyna Winnicka

**Affiliations:** 1 Department of Pharmaceutical Technology, Medical University of Białystok, Mickiewicza 2c, 15-222 Białystok, Poland; magda220796@gmail.com (M.D.); joanna.potas@umb.edu.pl (J.P.); kwin@umb.edu.pl (K.W.); 2 Unilever Polska Sp. z o.o.Al. Jerozolimskie 134, 02-305 Warsaw, Poland; katarzyna.woslatosi@unilever.com; 3 Adamed Pharma S.A., Preformulation Department R&D, Pieńków 149, 05-152 Czosnów, Poland; 4 Department of Biopharmaceutics and Pharmacodynamics, Medical University of Gdańsk, Hallera 107, 80-416 Gdańsk, Poland; julia.jacyna@gumed.edu.pl (J.J.); michal.markuszewski@gumed.edu.pl (M.J.M.)

**Keywords:** microparticles, chitosan glutamate, drug release, ion crosslinker, zidovudine

## Abstract

Microparticles containing water-soluble zidovudine were prepared by spray-drying using chitosan glutamate and beta-glycerophosphate as an ion crosslinker (CF). The Box–Behnken design was applied to optimize the microparticles in terms of their drug loading and release behavior. Physicochemical studies were undertaken to support the results from dissolution tests and to evaluate the impact of the crosslinking ratio on the microparticles’ characteristics. The zidovudine dissolution behavior had a complex nature which comprised two phases: an initial burst effect followed with a prolonged release stage. The initial drug release, which can be modulated by the crosslinking degree, was primarily governed by the dissolution of the drug crystals located on the microparticles’ surfaces. In turn, the further dissolution stage was related to the drug diffusion from the swollen polymer matrix and was found to correlate with the drug loading. Differential Scanning Calorimetry (DSC) studies revealed the partial incorporation of a non-crystallized drug within the polymer matrix, which correlated with the amount of CF. Although CF influenced the swelling capacity of chitosan glutamate microparticles, surprisingly a higher amount of CF did not impact the time required for 80% of the drug to be released markedly. The formulation with the lowest polymer:CF ratio, 3:1, was selected as optimal, providing satisfactory drug loading and displaying a moderate burst effect within the first 30 min of the study, followed with a prolonged drug release of up to 210 min.

## 1. Introduction

Chitosan, a biocompatible polycationic copolymer, is obtained from abundantly accessible chitin in a deacetylation process [1]. It consists of randomly ordered N-acetylglucosamine and glucosamine units linked by β (1 → 4) glycosidic bonds. Due to its cationic nature, chitosan binds readily to mucosal surfaces and thus has been applied to a number of medical and pharmaceutical preparations, including drug carriers [2,3], wound dressings [4], scaffolds for tissue engineering [5] and nano-carriers [6]. Chitosan has received considerable attention as an antifungal and antibacterial agent [7,8], as well as for being an antiviral adjunctive. Specifically, in vitro studies performed by the Russo group [9] showed that chitosan nanoparticles loaded with forscarnet exhibited a direct activity against the avian myelocytomatosis virus, whereas Artan et al. [10] found that sulfated chitosan oligomers were effective against the HIV virus. During our previous studies, we observed that chitosan in contact with vaginal fluid creates a swellable hydrogel matrix which may act as a barrier and support mucosal tissue against microbial infection [11].

With regard to buccal, nasal, and vaginal delivery carriers, chitosan or its derivatives appear to be promising, particularly because of their unique properties of penetration enhancement [12]. Despite the potential of chitosan in the technology of drug carriers for local application, its pH-dependent solubility and sensitivity to the presence of ions may lead to excessive water uptake. This in turn may cause a high initial burst release followed with the unwanted, fast disintegration of chitosan carrier in physiological fluids.

Among different methods to overcome the too-rapid dissolution of chitosan-based dosage forms and to assure a more controllable (prolonged or sustained) drug release profile, the modification of the polymer structure through mild physical crosslinking has been extensively studied [13,14]. In this process, which does not require catalysts or final product purification, a network of ionic bridges between negatively charged components (anions, ionic molecules, or negatively charged polymers) and polycationic chitosan is formed. We have previously shown that physical crosslinking between chitosan and beta-glycerophosphate (a non-toxic factor which enables interaction with chitosan under mild conditions) ensured the prolonged release of poorly water-soluble antifungal drugs [15].

This study aims at investigating the influence of microparticle composition, in particular the amount of introduced beta-glycerophosphate as an ion crosslinker (CF) on the release profile of water-soluble drugs from the chitosan matrix. For this purpose, spray-dried microparticles composed of chitosan glutamate (gCS) with a potential as a microbicide vaginal delivery platform were chosen [16]. Zidovudine (ZVD)—an antiretroviral agent classified as freely water-soluble (solubility 26-50 mg/mL at ambient temperature, pKa 9.53) was selected as the model active agent. Regarding the fact that spray-drying involves complex interactions between process factors and the final formulation characteristics, a design of experiments (DoE) approach was implemented to optimize the gCS-based microparticles in terms of drug loading and its release behavior and to understand the relationship between the input parameters (including inlet temperature, polymer:CF and polymer:drug ratio) and product quality attributes. A broad physicochemical analysis was additionally carried out for the selected formulations to support the data from the dissolution studies.

## 2. Materials and Methods

### 2.1. Materials

The highly purified medical grade gCS (Chitoscience^®^) with an individual certificate of analysis was from Heppe Medical Chitosan GmbH (Halle, Germany). The precise deacetylation degree (79.5%) was assessed by the titration method [17]. The zidovudine in crystalline form (batch ZD1900515, impurities level 0.16%) was donated by Hetero Labs Limited (Baddi, India). Methanol with a HPLC grade was obtained from Merck (Darmstadt, Germany), whereas the beta-glycerophosphate (≥99% pure) was from Sigma Aldrich (Darmstadt, Germany). The water for the HPLC was distilled and passed through a Milli-Q Reagent Water System (Billerica, MA, USA). The simulant vaginal fluid (pH 4.2) used for the in vitro release and swelling tests was composed of (in g): sodium chloride, 3.51; potassium hydroxide, 1.40; calcium hydroxide, 0.222; bovine serum albumin, 0.018; lactic acid, 2.00; acetic acid, 1.00; glycerol, 0.16; urea, 0.4; glucose, 5.0; and water up to 1000 mL [18]. Other chemicals with an analytical purity grade were from Chempur (Piekary Śląskie, Poland).

### 2.2. Spray-Drying Technique

The microparticles were produced using a Buchi Mini Spray Dryer B290 (Flawil, Switzerland) according to the method previously described by our group [15]. In brief, 2% (*w/w*) gCS aqueous dispersion was prepared by dissolving polymer at 30 °C. An appropriate amount of ZVD was dispersed in water with an addition of 5% of ethanol as a cosolvent and carefully combined with polymer dispersion under continuous stirring. All the tests were carried out in an opened loop configuration at a spray rate of 1.2 mL/min. The aspirator blower capacity was 95%, whereas the pressure was kept at 40 mm Hg in all experiments. The inlet temperature (T_in_) was in the range of 110–130 °C, whereas the outlet temperature varied between 68 °C and 80 °C.

### 2.3. Microparticles Optimization

A Box–Behnken experimental design consisting of 15 experiments, including 3 replications of the center point was used in the optimization process of the gCS-based microparticles in terms of the ZVD loading and release profile. Based on previously performed screening studies with the use of Fractional Factorial Design [16], an established design space for input factors (namely, an inlet temperature in the range of 110–130 °C and a gCS:ZVD ratio ranging from 2 to 3 (*w/w*)) was provided. Furthermore, an additional factor, namely the gCS:CF ratio, varying in the range of 3:1 to 1:1 was included in experiments. An experimental plan was created to predict the technological settings, ensuring targeted microparticles quality parameters using JMP software (version 8.0, SAS Institute, Cary, NC, USA). The process variables differed in accordance with the experimental design matrix (Table 1). A placebo formulation with 2% gCS concentration and a gCS:CF ratio at 2:1 prepared at 130 °C was obtained as an additional control.

### 2.4. Dissolution Studies

In vitro release studies were carried out in a USP II apparatus (Agilent 708-DS, Agilent Technologies, Cary, NC, USA) equipped with Teflon enhancer cells (total diffusion area: 3.8 cm^2^). Each microparticles sample (which referred to 10 mg of the drug) was located in an enhancer cell and covered with a prehydrated Cuprophan membrane (the molecular weight cut off (MWCO): 10000Da; Medicell, London, UK) to reflect the conditions of the vaginal cavity more appropriately [19]. Simulated vaginal fluid (pH 4.2) was used as the dissolution medium (100 mL). The stirring rate was 75 rpm and the temperature was maintained at 37 ± 0.5 °C. The acceptor medium (2 mL) was withdrawn at the determined time points, diluted with the medium, spectrophotometrically quantified at 265 nm (Hitachi U-1800, Tokyo, Japan), and compared to the standard solution. An equal volume of the acceptor medium replaced the withdrawn samples. All the dissolution measurements were performed at least in triplicate.

### 2.5. Microparticles Characterization

#### 2.5.1. Drug and Moisture Content

The ZVD content was assessed by drug extraction from microparticles using a mixture of water and methanol (4:1, *v/v*) at 30 °C (agitation 200 rpm, 3 h). After filtration through 0.45 µm nylon filter (Millipore, Billerica, MA, USA), the drug concentration was assessed by the HPLC technique, as defined in Section 2.6.

The moisture content was determined by heating the microparticles sample up to 120 °C using the moisture analyser Radwag WPS 50SX (Radom, Poland).

#### 2.5.2. SEM Analysis

A Carl Zeiss EVO/LS25 scanning electron microscope (Carl Zeiss AG, Oberkochen, Germany) equipped with a backscattered electron detector was applied to examine the surface morphology of the selected formulations. The samples were mounted on an aluminum stage using adhesive carbon tape and located in the microscope chamber. SEM was operating in a variable pressure mode at nitrogen gas pressure in the range 70–90 Pa at an accelerating voltage of 20 kV.

#### 2.5.3. Particle Size Distribution (PSD)

The particle size distribution of the selected formulations was determined by an automatic optical light microscope, Morphologi G3 (Malvern Panalytical, Malvern, UK), using the static image analysis method. All the experiments were conducted using a50x NA 0.8 objective and episcopic sample illumination. Each sample of microparticles was suspended in a 1% lecithin solution in octanol and sonicated for 30 sec. The obtained suspension was placed on a glass slide, left to air dry and immediately analyzed. From each measurement, the particle size (expressed as volume and number distribution) was plotted against a logarithmic-scaled Circle Equivalent (CE) diameter expressed in µm. The values of D_10_, D_50,_ and D_90_ representing the CE diameters below which lay respectively 10%, 50%, and 90% of the distribution were designated for both the number and volume PSD plots.

#### 2.5.4. Differential Scanning Calorimetry (DSC)

Differential scanning calorimetry (DSC) measurements of the ZVD, gCS, placebo microparticles, and selected ZVD-loaded formulations were conducted on the Mettler-Toledo Star 1 (Greifensee, Switzerland). About 0.5–1 mg of the samples were placed in sealed aluminum pans with pierced lids. A simple heating program (from 30 °C to 165 °C, with a gradient heating rate of 10 °C/min) under a dry nitrogen atmosphere (nitrogen flow 30 mL/min) was applied in each run. The degree of crystallinity of ZVD in the selected microparticles’ formulations was determined by using the crystalline ZVD melting enthalpy ΔH_100%_ as a reference value. For this purpose, physical blends of the crystalline ZVD and placebo formulation were prepared in mass ratios of 1:10, 2:10, and 3:10 (n = 3). For the selected ZVD-loaded formulations, the melting enthalpy ΔH_MP_ (expressed in J/g of ZVD active substance) was determined from three consecutive measurements, one for each sample, and calculated in respect to each drug loading assessed with the HPLC method. The degree of crystallinity was expressed as a percentage value (%DC) and then calculated as a ratio of ΔH_MP_ for a given ZVD-loaded formulation to ΔH_100%._

#### 2.5.5. Raman Confocal Spectroscopy

An Alpha300R confocal Raman microscope (WITec, Ulm, Germany) was used to create a cross-section surface map of the selected ZVD-loaded formulation. For this purpose, a 785 nm diode laser (power 120 mW) was used in combination with a 300 g/mm, BLZ 750 nm grating, a True Surface attachment for the microscope *z*-axis correction, and a Epiplan-Neofluar 50× NA 0.8 objective (Carl Zeiss, United Kingdom). A map 100 µm × 100 µm (150 px × 150 px) in size was collected with a spatial resolution of approximately 2 µm. Phases corresponding to the ZVD and microparticle matrix (gCS and CF as a single phase) on the analyzed surface were identified by using statistical means—a basis analysis, spectra demixing, and comparison with the previously acquired reference spectra of the analyzed components.

#### 2.5.6. X-Ray Powder Diffraction (XRPD)

XRPD was applied to determine the solid state of the pure drug and the selected formulation of microparticles using a Empyrean Series 3 (Malvern Panalytical Ltd., Eindhoven, Netherlands), equipped with Bragg–Brentano θ–θ geometry goniometer (240 mm radius), PIXCel^1D^ detector, and a ceramic X-ray tube with a copper anode at 45 kV and 40 mA (K_α1_ equal 1.54060 Å) as an X-ray source. Each sample was scanned in the range of 2° to 40° 2θ, with a step size of 0.007° 2θ and a scan step time of 13.77 s.

#### 2.5.7. Swelling Capacity Studies

The swelling capacity was performed using a modified Enslin apparatus, as previously described [20]. A certain amount of the selected formulations was spread homogeneously on the cellulose filter paper soaked with simulant vaginal fluid (SVF), pH 4.2. The amount of liquid absorbed by the microparticles was measured within 240 min. All the experiments were carried out in triplicate and expressed as the percentage of fluid uptake vs. the initial weight of the formulations’ samples [15].

### 2.6. HPLC Analysis

A HPLC system (Agilent Technologies 1200, Waldbronn, Germany) equipped with a UV-Vis detector G1315B, binary pump G1312A, degasser G1379B, and thermostat G1316A was applied to determine the ZVD. Basically, separation was attained on the Zorbax Eclipse XDB–C18, 250 mm × 4.6 mm, 5 μm column (Perlan Technologies, Warsaw, Poland), according to the method previously described by [21]. The analysis was carried out at 30 °C with the mobile phase consisting of a methanol and acetic buffer, pH 4.5 (30:70, *v/v*), at a flow rate of 1.0 mL/min. UV detection was achieved at 265 nm. The linearity of the calibration curve was calculated using a linear least-squares regression analysis of the drug concentration vs. the plot of peak area. The obtained calibration curve was found to be linear, in the range of 2–50 μg/mL (*R*^2^ = 0.991).

### 2.7. Statistical Analysis

The quantitative variables were expressed as the mean ± standard deviation (SD) by MS Excel software. The measurements were considered significant, at *p* < 0.05.

## 3. Results and Discussion

### 3.1. Dissolution Studies and Microparticles Optimization

In the present work, the effect of microparticles composition on their characteristics and the release profile of water-soluble ZVD was investigated using DoE, a statistical tool related to the quality by design approach extensively applied in pharmaceutical technology at the stage of product development, process optimization, or validation. Generally, DoE can be divided into screening and optimization techniques, which are selected with respect to the defined object, the number of input parameters and interactions to be tested, and the effectiveness of the design or statistical validity [22].

In order to optimize the microparticles in terms of their drug loading and dissolution profiles, a Box–Behnken experimental design was applied. The objective was to obtain a prolonged drug release from the polymer matrix with a simultaneous moderate initial burst effect. Such release behavior with the assurance of rapid drug activity over an extended period of time agrees with the vaginal microbicide strategy to prevent sexually transmitted infections [23]. Apart from different gCS:CF ratios, the content of ZVD and gCS:ZVD ratio as well as the inlet temperature were chosen as variables with a plausible effect on the drug dissolution profile from the microparticles matrix. As we previously observed that polymer concentration played a substantial role in controlling the degree of swelling and the drug release rate, we decided to exclude the impact of this parameter and apply one concentration of gCS (2%, *w/w)* in these studies.

Overall, as a result of 15 experiments, the microparticles differing in gCS:ZVD and gCS:CF ratios were spray-dried, with a production yield the range of 50–86%. All the formulations took the form of relatively free-flowing white powder in which the moisture content did not exceed 7% regardless of the applied process temperature. Drug loading was found in the range between 14.3% (M3) and 25.3% (M14) (Table 2). Macroscopically, the microparticles with a higher gCS:ZVD ratio (3:1, *w/w*) and simultaneous greater crosslinking degree (gCS: CF 1:1, *w/w*) were slightly more adhesive. The presence of higher amounts of ZVD and lower CFs was found to improve the particles’ flowability.

**Figure 1:**
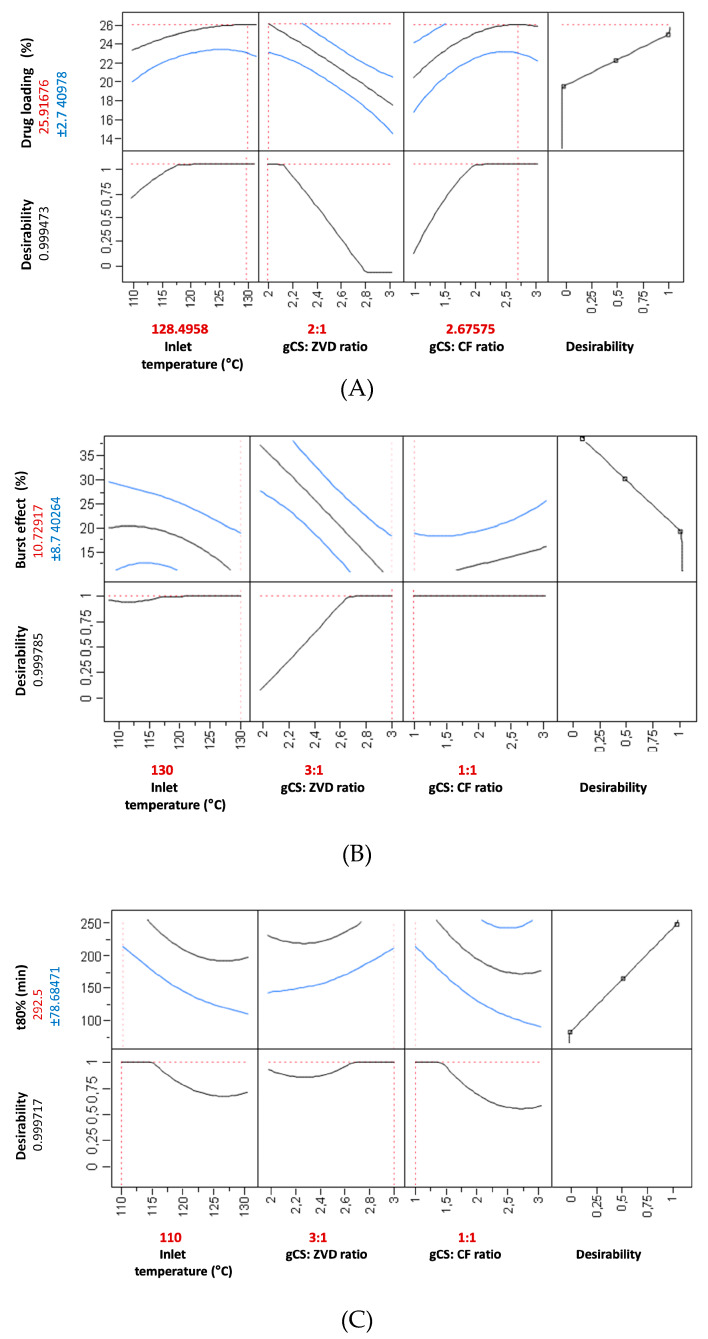

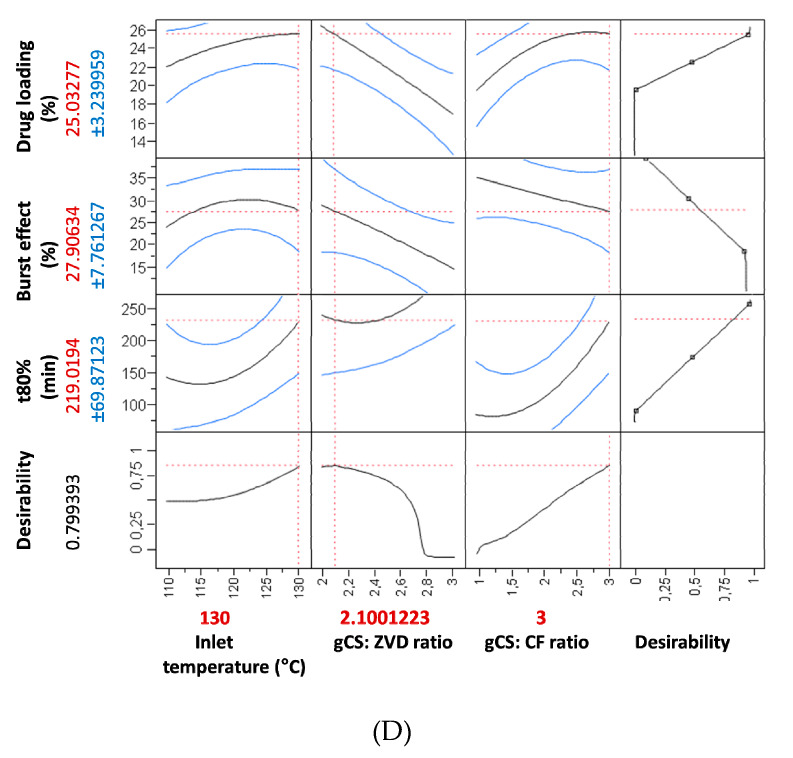
Predicted optimal factors settings (in red) for each studied response, with the use of a maximized desirability function. (**A**–**C**)—the individual optimization of each factor, (**D**)—the simultaneous optimization of all the studied factors.

The obtained results (measurements of selected responses) from the experimental design were used to build separate models, in order to show which input factors settings ensured their desired values (Figure 1). Such an approach enabled us to predict the influence of each tested parameter on the microparticles’ characteristics. Critical values were introduced into each model. In brief, undesirable responses (for which the “desirability” was established as 0) were: a ZVD loading of ≤ 20%, a burst effect (expressed as the % of drug released within the first 30 min) of ≥ 40%, the time required for 80% of drug release (t80%) ≤ 90 min. Whereas, the expected values (desirability equaled 1) were set up as follows: a ZVD loading of ≥ 25%, a burst effect of ≤ 20%, and a t80% of ≥ 210 min. All the obtained models were found of statistical significance, with a *p* value ranging from 0.008 to 0.043.

An increase in the inlet temperature had a positive effect on the ZVD loading. A higher amount of ZVD (gCS:ZVD ratio 2:1, *w/w*) improved the loading capacity, whereas the presence of CF was found to exert a slightly negative effect on the drug entrapment in the polymer matrix (Figure 1A).

**Figure 2:**
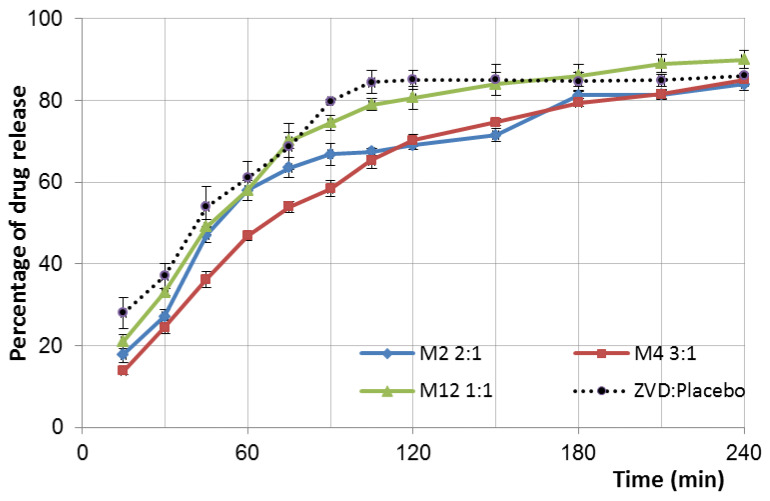
In vitro Zidovudine (ZVD) release from microparticles M2, M4, and M12 differed in chitosan glutamate (gCS):CF ratios compared to the ZVD blend with placebo microparticles (gCS:CF 2:1, *w/w*), displaying the impact of the ion crosslinker (CF) ratio on the dissolution profile (mean ± SD; *n* = 3).

The gCS/CF microparticles exhibited prolonged ZVD release rates when compared to the control: ZVD mixed with placebo formulation (Figure 2). The simple blend of ZVD with placebo microparticles composed of gCS and CF at a ratio of 2:1 (*w/w*) was found to be incapable of modulating the drug release, as the burst effect exceeded 35% and the t80% was achieved within 90 min of studies. Two stages were noticed during the drug release from microparticles: an initial burst release and a subsequent prolonged release phase (Figure 2). Profound differences in the ZVD dissolution pattern were observed among the formulations. The time required for 80% of drug release varied from 90 to up to 210 min. The formulation with gCS:ZVD 2:1 and gCS:CF 2.5:1 (experimental domain center point M7, M8, and M11) demonstrated the fastest release rate among the tested formulations (Table 2). In contrast, the microparticles M3 and M4 were found to exhibit a prolonged dissolution rate of up to 210 min, with simultaneous values of the burst effect below 25% (Table 2).

Based on the individual models, it was found that each studied response would benefit from extremely different conditions. However, the simultaneous optimization of all three examined responses (where the studied parameters were equally important for the model) enabled us to find mutually optimal parameter settings. The suggested parameter settings, similar to the formulation of M4 characterized by optimal properties, would be an inlet temperature of 130 °C, a gCS:ZVD ratio equal to 2.1, and a gCS:CF ratio set at 3 (Figure 1D). The most significant factors in the obtained model were the gCS:ZVD and gCS:CF ratios (FDR *p* values 0.0029 and 0.0462, respectively). Although the inlet temperature was not found to be statistically significant, this factor was found to be important for the optimization of the process through interaction with the gCS:ZVD ratio (FDR *p* value 0.0389).

In brief, a decrease in the gCS:ZVD ratio was found to be responsible for a higher initial drug release (Figure 1B). The lowest applied gCS:ZVD ratio (2:1, *w/w*), and in consequence a higher drug loading (results above 20%), was responsible for a more pronounced burst effect (above 25%) from the gCS/CF matrix. The analysis also displayed an impact of the crosslinking ratio on the initial release behavior. A relatively reduced burst effect was noticed for the samples with the highest gCS:CF ratio of 1:1 (e.g., M3, M10, M15). Surprisingly, these trends may vary depending on the settings of the other two parameters tested, suggesting the presence of higher order interactions (Figure 1B,D).

The DoE approach revealed that the crosslinking ratio had a moderate influence on the overall dissolution rate t80%. Nevertheless, it is worth adding that the influence of each variable depends on the settings of the other parameters, which might be observed when comparing the drug release patterns from selected formulations differing in settings of one parameter but identical in terms of the other variables. For example, the formulations M4 and M15 (differing in crosslinking ratio; Table 2) provide a 60 min difference in t80% in favor of formulations with a lower amount of CF, but formulations M10 and M13 (prepared using a lower T_in_) indicate the opposite trend. This set of experiments extends our previous observations on ion crosslinked chitosan hydrogels, in which a markedly lower dissolution rate was noticed for formulations with a higher concentration of crosslinker [11]. Similarly, several other research papers described a profound impact of ion crosslinker on the drug release behavior from chitosan drug delivery systems, mostly as a result of the formation of more intact structures in the dissolution media [14,24,25].

### 3.2. Morphological and Physicochemical Characterization of gCS/CF Microparticles

A morphological and physicochemical analysis was carried out to support the data from the dissolution studies and investigate the influence of ion crosslinking on the microparticle characteristics. For this purpose, the microparticles M2, M4, and M12 varying in the gCS:CF ratio (and simultaneously comparable drug loading values) were selected based on the experimental design and obtained dissolution results. Additional samples with an extreme ZVD load (M3 and M5) were added to the tested pool.

**Figure 3:**
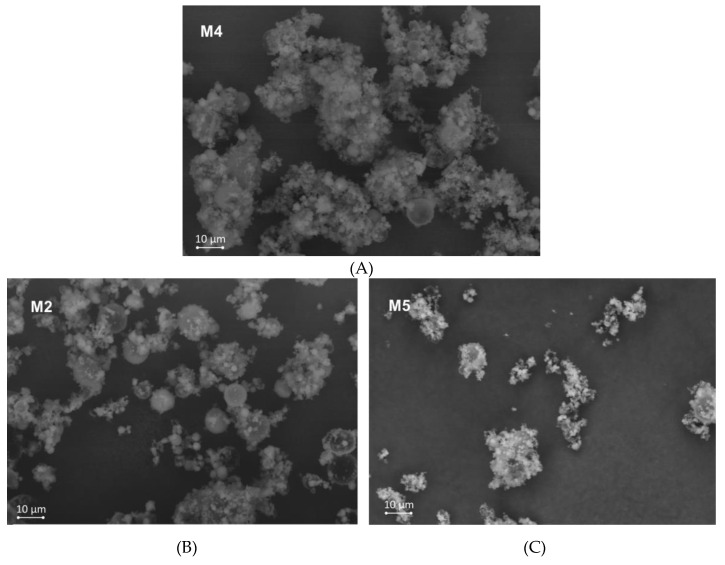

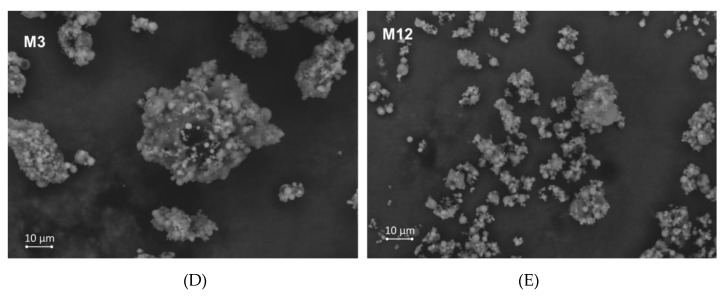
SEM images of zidovudine (ZVD)-loaded microparticles with chitosan glutamate and beta-glycerophosphate disodium in a ratio 3:1: M4 (**A**); 2:1: M2 (**B**) and M5 (**C**); 1:1: M3 (**D**) and M12 (**E**); original magnification ×2000.

The morphological examination performed with scanning electron microscopy displayed the predominant presence of spherical structures (Figure 3). Basically, all the tested microparticles displayed a relatively homogenous surface morphology, with the presence of drug crystals on the gCS/CF surface (in contrast to the placebo formulation presented in Figure S1). The samples varied in respect to the microparticle size and size uniformity. Sample M12 exhibited a reduced rate of agglomeration and no presence of coalesced particle structures, which can be correlated with a higher ZVD content and a simultaneous high crosslinking degree in the formulation (Figure 3E). In contrast, all the other tested formulations (Figure 3A–D) had a pronounced tendency to form agglomerates, which might be attributed to more pronounced electrostatic inter-particles forces.

**Figure 4:**
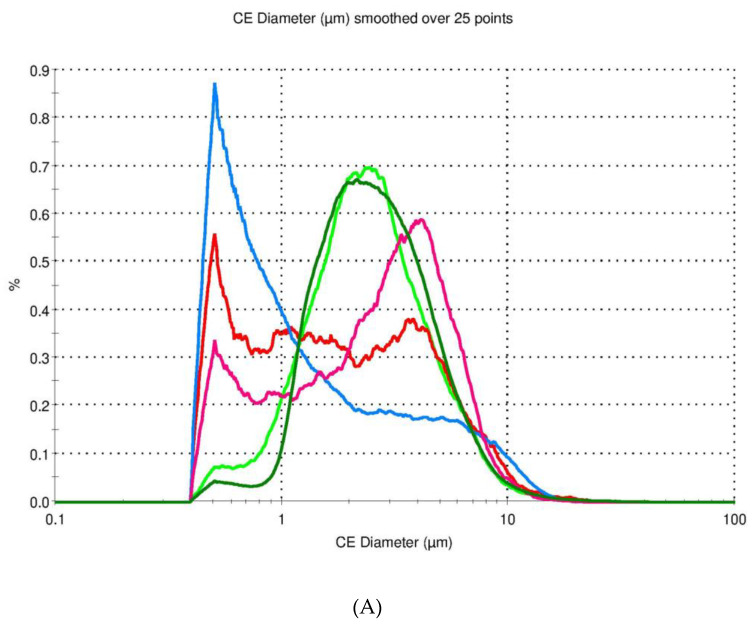

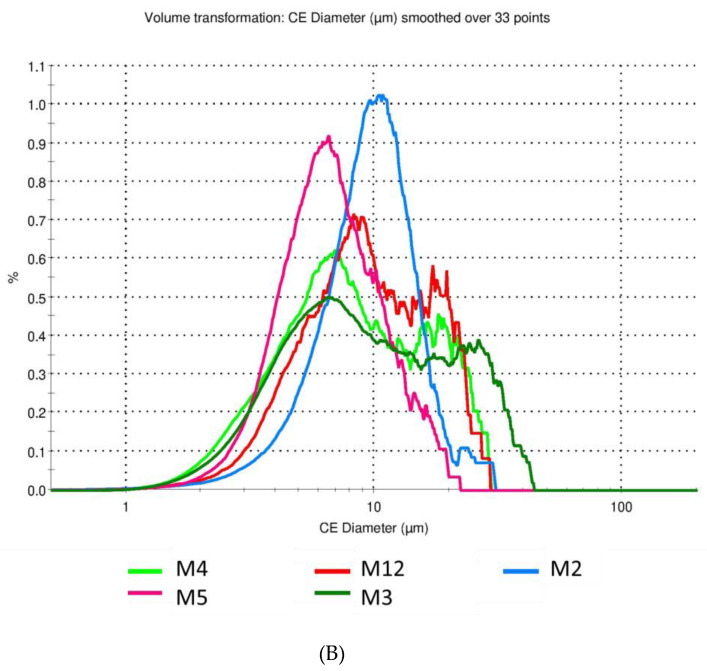
Number (**A**) and volume (**B**) size distribution of the selected gCS/CF microparticles with zidovudine.

The particle size distributions (expressed as a number and volume distribution) of the selected formulations M2, M3, M4, M5, and M12 varying in gCS/CF and gCS:ZVD ratios are displayed in Figure 4 and Table 3.

All the tested samples displayed a narrow number size distribution in contrast with the results from the volume distribution, in which particles with diameters over >20 µm were visible (Table 3). The transformation of results from a number to volume-based distribution caused a profound alteration in the median (D_50_) range from 1.0–2.7 μm to 6.5–9.7 μm.

The results from the number distribution marked out the formulations M3 and M4 as the ones with the lowest number of particle fraction <1 µm CE diameter and a shape close to Gaussian distribution (Figure 4A). In turn, the microparticles M2, M5, and M12 had a distinct below 1 µm CE diameter fraction and bimodal distribution. This finding can be correlated with SEM observations that revealed coalesced particles. Although the sample preparation included sonification, which split the agglomerates held by electrostatic forces into individual particles, the coalesced structures were left intact and subsequently did not produce fine fractions for the M3 and M4 formulations. The presence of a higher fraction of smaller microparticles (with a large surface area to volume ratio) could have been responsible for speeding up the drug release.

A particle size analysis revealed bimodality also for the volume size distribution, particularly evident for the formulations M3, M4, and M12, whereas the M2 and M5 samples exhibited close to Gaussian distributions (Figure 4B). The predominance of particle fractions with a CE diameter > 20 µm was mostly responsible for the volume distribution’s bimodal shape. The described effects can be correlated with differences between the studied formulations. An increase in the gCS:CF ratio from 2:1 to 1:1 *w/w* increased the particle size D_90_ (M3 vs. M5 and M2 vs. M12). When comparing formulations with the same gCS:ZVD ratio, a lower amount of encapsulated ZVD slightly shifted the size distribution to higher values (M3 vs. M4, with a gCS:ZVD ratio of 3:1 and 2:1 *w/w,* respectively). Formulation M4 was the most homogenous in terms of size distribution among the tested samples, with a distinct fraction of microparticles (above 1 µm) which may contribute to the prolonged drug dissolution rate (Figure 2).

**Figure 5:**
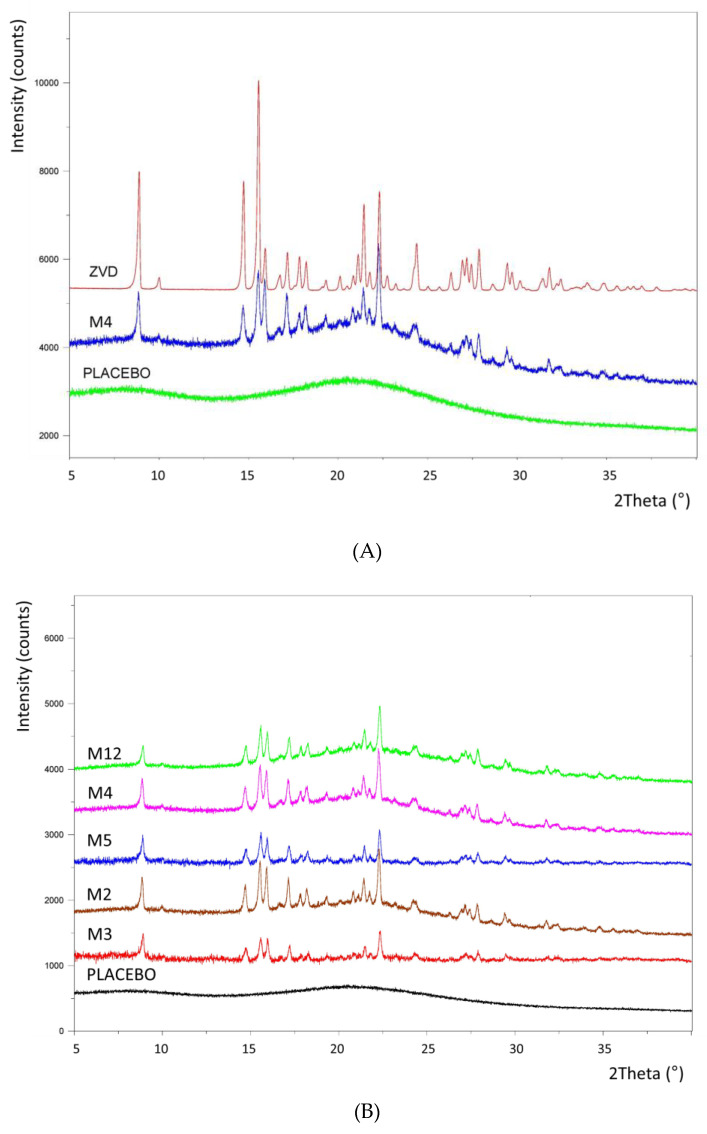
X-Ray Powder Diffraction (XRPD) patterns of (**A**) pure zidovudine (ZVD), placebo microparticles with chitosan glutamate crosslinked with β-glycerophosphate (gCS:CF 2:1, *w/w*), and the formulation M4 (gCS:CF ratio 3:1, *w/w*); (**B**) selected drug-loaded formulations differed in their crosslinking ratio (M2 2:1; M3 1:1; M4 3:1; M5 2:1; M12 1:1, *w/w*).

ZVD is known to have a single crystalline form [26] with a characteristic X-ray diffraction pattern (Figure 5). The results of the XRPD study conducted for the selected microparticle formulations (M2, M3, M4, M5, M12) enabled the identification of one ZVD crystalline form in all the tested samples (Figure 5B). This finding showed that the spray-drying manufacturing procedure at least partially produced the active ingredient in its crystalline form.

**Figure 6:**
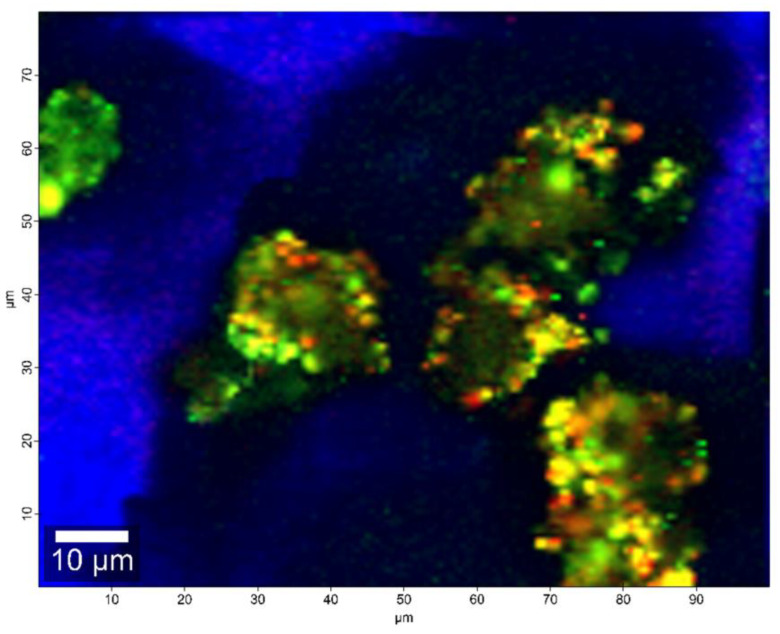
Raman surface distribution map of microparticle formulation M4: ZVD (red), gCS/CF matrix (green), ZVD- gCS/CF mixed regions (yellow), glass surface (blue).

A Raman confocal microscopy analysis illustrated the surface distribution of the ZVD and gCS/CF matrix (combination of gCS and CF as a single component) on the selected M4 formulation sample (Figure 6). The following regions can be distinguished: areas of high ZVD surface concentration (single component—red Figure 6), areas where the gCS/CF matrix only was found (single component—green), and mixed areas containing both ZVD and gCS/CF in moderate concentrations (yellow). These observations indicated that the active substance is not evenly distributed within the gCS/CF matrix volume. Subsequently, there is a possibility that ZVD may exist in crystalline form only partially as an excess on the microparticles’ external surfaces. ZVD encapsulated within the matrix volume might exhibit different physical interaction and a non-crystalline arrangement that would not contribute to the obtained X-ray diffraction pattern.

**Figure 7:**
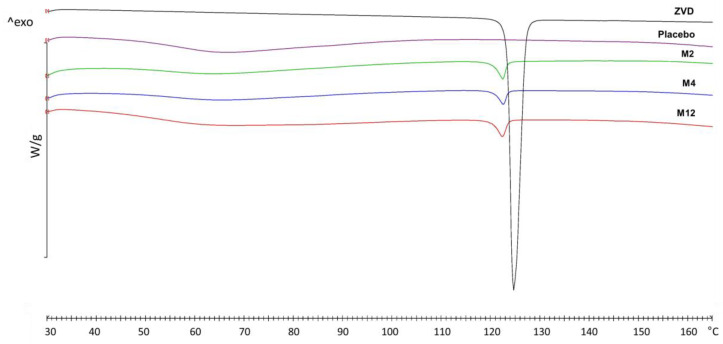
Thermograms of pure zidovudine (ZVD), placebo microparticles with chitosan glutamate crosslinked with beta-glycerophospahe (gCS:CF 2:1), and selected drug-loaded formulations differed in the crosslinking ratio gCS:CF (M2 (2:1); M4 (3:1); M12 (1:1), *w/w*).

DSC experiments were performed to determine the semi-quantitative amount of crystalline ZVD in the selected formulations. The DSC thermograms of all microparticles in coherence with the obtained XRPD results exhibited an endothermic peak, with an onset occurring at 120–123 °C corresponding with the ZVD melting point (Figure 7). Enthalpy associated with this characteristic transition was utilized to assess the amount of crystalline ZVD fraction in the tested formulations. In order to evaluate the degree of crystallinity (%DC) in the microparticles, blends containing crystalline ZVD and a placebo formulation with gCS:CF 2:1, *w/w* were used as a reference (Table 4).

As is shown in Table 4, profound differences in the amount of crystalline ZVD among the tested formulations were noticed, with the highest total ZVD load of approximately 50% for samples M2 and M5. These findings are in correspondence with observations from the Raman analysis, indicating that a recrystallized ZVD was not the only physical form of drug incorporated within the gCS:CF matrix volume. A correlation between the gCS:CF ratio and the ZVD degree of crystallinity in the examined formulations was observed. Basically, the drug-loaded microparticles with the highest crosslinking degree (gCS:CF ratio 1:1, *w/w*) possessed lower values of % DC, which may suggest that a higher crosslinking degree contributed to the greater incorporation of ZVD in its non-crystalline state within the microparticle volume. This observation also indicates that some kind of interaction between ZVD and the gCS/CF matrix might have been responsible for the modulation of the drug release pattern after the spray-drying process (Figure 2, Table 2).

**Figure 8:**
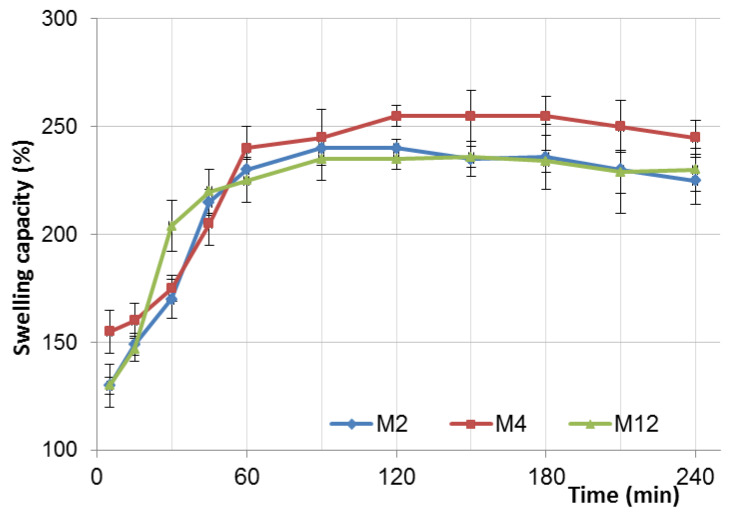
Swelling index study of the selected microparticle formulations with a ratio gCS:CF of 1:1 (M12), 2:1 (M2), 3:1 (M4) (mean ± SD; *n* = 3).

Regarding the fact that drug release from chitosan preparations is usually associated with its swelling capability, the selected microparticles were also subjected to a water-uptake test. As is displayed in Figure 8, all the tested formulations showed a swelling capacity, which assessed the highest increment within the first 60 min of the studies.

Complete hydration and dissolution (with the simultaneous formation of a viscous polymer matrix) was observed after 90 min (M2, M12) and 120 min (M4). The influence of the crosslinking ratio on the swelling process was shown, and the microparticle M12 (with a gCS:drug ratio of 1:1) demonstrated the lowest hydration ability. Unexpectedly, this formulation exhibited the highest water-uptake capacity within the first 30 min. Most likely, an observed initial enhanced hydration of ion-crosslinked gCS microparticles enabled easier drug diffusion from the polymer matrix upon contact with SVF, which might expedite the drug release rate (Figure 2). In contrast, M4 (gCS:CF, 1:1, *w/w*) had a lower degree of swelling at the same time, which could hinder the initial drug release from the matrix and diminish the rate of the burst effect.

## 4. Conclusions

As a result of the optimization process, the formulation M4 with a gCS:ZVD ratio of 2:1, a gCS:CF ratio set at 3:1, and that was spray-dried at 130 °C displayed high drug loading and the desired drug release profile. The obtained findings point to the complex nature of water-soluble ZVD release from gCS/CF, which depends either on the amount of encapsulated drug, the degree of crosslinking, or physical properties of the spray-dried microparticles. Although every tested gCS-drug-CF combination exhibited its own release profile, some patterns and correlations could be distinguished. In formulations with a higher ZVD content, desorption and dilution seemed to be a controlling factor resulting from an excess amount of recrystallized drug located on the microparticles’ surfaces. In turn, the drug in a non-crystallized state (which was found to be at least partially dependent on the crosslinking degree) more strongly bound to the gCS/CF matrix, which resulted in a decrease in the release rate. Although a clear impact of the crosslinking degree on the initial burst effect and swelling capacity was observed, the crosslinking ratio surprisingly did not exert a pronounced influence on the time required for 80% of the drug dissolved from the gCS microparticles. The effect of the physical characteristics of microparticles on the release process was also visible. For instance, the presence of a higher fraction of smaller microparticles (with a large surface area to volume ratio) could speed up the drug release rate. Thus, further optimization studies are still needed in terms of attaining a greater amount of non-crystallized drug within the polymer matrix.

## Figures and Tables

**Table 1:** The studied variables and certain level settings in the applied Box–Behnken design.

Formulation	Pattern *	gCS:CF Ratio (*w/w*)	gCS:ZVD Ratio (*w/w*)	T_in_ (°C)
M1	0+−	2:1	3:1	110
M2	0−−	2:1	2:1	110
M3	−+0	1:1	3:1	120
M4	+0+	3:1	2.5:1	130
M5	0++	2:1	3:1	130
M6	++0	3:1	3:1	120
M7	000	2:1	2.5:1	120
M8	000	2:1	2.5:1	120
M9	0−+	2:1	2:1	130
M10	−0−	1:1	2.5:1	110
M11	000	2:1	2.5:1	120
M12	−−0	1:1	2:1	120
M13	+0−	3:1	2.5:1	110
M14	+−0	3:1	2:1	120
M15	−0+	1:1	2.5:1	130

* In order to estimate influence of 3 parameters, 15 experiments, including 3 center points (Pattern 000), were carried out in a randomized order. Pattern—encoded description of factor settings in subsequent experiments.

**Table 2:** Drug loading and results from dissolution studies expressed as the percentage of drug released within the first 30 min (burst effect) and the time required for 80% of the drug dissolved in the acceptor medium (t80%) (*n* = 3).

Formulation	gCS:CF Ratio (*w/w*)	gCS:ZVD Ratio (*w/w*)	ZVD Loading * (%)	Burst Effect * (%)	t80% (min)
M1	2:1	3:1	17.5 ± 0.3	25.9 ± 2.0	210
M2	2:1	2:1	23.1 ± 2.0	26.0 ± 2.1	180
M3	1:1	3:1	14.3 ± 0.3	20.3 ± 1.9	210
M4	3:1	2.5:1	22.3 ± 0.6	23.4 ± 1.1	210
M5	2:1	3:1	18.1 ± 1.1	15.1 ± 1.2	180
M6	3:1	3:1	16.3 ± 1.2	26.4 ± 1.9	210
M7	2:1	2.5:1	22.6 ± 1.5	25.9 ± 1.3	105
M8	2:1	2.5:1	20.5 ± 0.8	27.4 ± 1.8	90
M9	2:1	2:1	24.3 ± 0.9	32.7 ± 1.9	105
M10	1:1	2.5:1	17.8 ± 1.3	21.4 ± 2.1	180
M11	2:1	2.5:1	21.9 ± 0.4	31.0 ± 1.7	90
M12	1:1	2:1	22.6 ± 0.7	33.1 ± 2.2	120
M13	3:1	2.5:1	19.6 ± 0.9	30.4 ± 2.0	120
M14	3:1	2:1	25.3 ± 1.0	29.8 ± 1.8	150
M15	1:1	2.5:1	16.8 ± 0.7	22.0 ± 2.4	150

* mean ± SD.

**Table 3:** Number and volume size distribution characteristics of the selected gCS/CF microparticles with zidovudine (ZVD).

Formulation	gCS:CF Ratio (*w*/*w*)	gCS:ZVD Ratio (*w*/*w*)	Number Distribution (µm)			Volume Distribution (µm)		
			D_10_	D_50_	D_90_	D_10_	D_50_	D_90_
M2	2:1	2:1	0.5	1.0	5.6	5.2	9.7	15.4
M3	1:1	3:1	1.3	2.5	5.2	3.6	8.9	26.4
M4	3:1	2.5:1	1.1	2.3	5.1	3.3	7.7	20.3
M5	2:1	3:1	0.6	2.7	5.8	3.7	6.5	11.9
M12	1:1	2:1	0.5	1.6	5.6	4.3	9.2	19.7

**Table 4:** Differential Scanning Calorimetry (DSC) results of the ZVD-loaded microparticles with chitosan glutamate crosslinked with beta-glycerophosphate (gCS/CF).

Formulation	gCS:CF Ratio (*w*/*w*)	ZVD ΔH_MP_ [J/g]	%DC *
**M2**	2:1	52.9 ± 1.2	51
**M3**	1:1	21.1 ± 0.5	21
**M4**	3:1	45.9 ± 1.6	45
**M5**	2:1	54.0 ± 1.0	52
**M12**	1:1	38.0 ± 0.1	37

* Degree of crystallinity established based on the enthalpy achieved for ZVD (ΔH_100%_ = 102.9 ± 7.1 J/g).

## Supplementary Materials

The following are available online at https://www.mdpi.com/1999-4923/12/5/455/s1, Figure S1: SEM image of zidovudine-free (placebo) microparticles with chitosan glutamate and beta-glycerophosphate disodium in a ratio of 2:1 (*w/w*).
