# Supplementary material for: The Correlation between Physical Crosslinking and Water-Soluble Drug Release from Chitosan-Based Microparticles
Source: Pharmaceutics. 2020 May 16;12(5):455. doi: 10.3390/pharmaceutics12050455 (PMC7284795; doi:10.3390/pharmaceutics12050455)
Supplement: Supplementary file 1 [file pharmaceutics-12-00455-s001.pdf]

pharmaceutics

Supplementary Materials: The Correlation between Physical Crosslinking and Water-Soluble Drug Release from Chitosan-Based

Emilia Szymańska, Katarzyna Woś-Latosi, Julia Jacyna, Magdalena Dąbrowska,
Joanna Potaś, Michał Jan Markuszewski and Katarzyna Winnicka

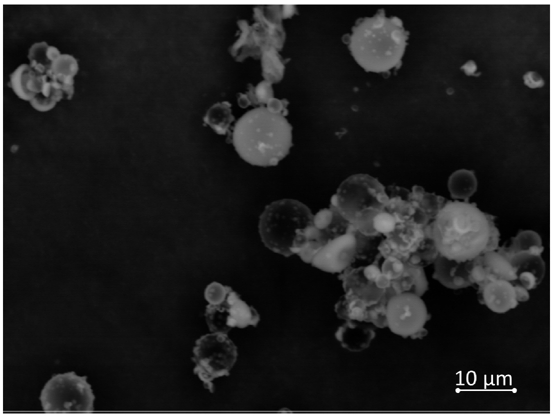

Figure S1. SEM image of zidovudine- free microparticles with chitosan glutamate and beta-glycerophosphate disodium in ratio 2:1; original magnification ×2000.
